# Absolute and relative disparity mechanisms revealed by an equivalent noise analysis

**DOI:** 10.1038/s41598-024-57406-2

**Published:** 2024-03-22

**Authors:** Jian Ding, Hilary H. Lu, Dennis M. Levi

**Affiliations:** Herbert Wertheim School of Optometry and Vision Science and the Helen Wills Neuroscience Institute, University of California, Berkeley, Berkeley, CA 94720-2020 USA

**Keywords:** Neuroscience, Psychology

## Abstract

The precision of stereopsis and vergence are ultimately limited by internal binocular disparity noise. Here we propose an equivalent noise model with both global and local internal disparity noises to provide a unified explanation of both absolute and relative disparity thresholds. To test this model, we developed a psychophysical procedure to measure the equivalent internal disparity noise by adding external disparity noise to random-Gabor-patch stereograms. We used the method of constant stimuli to measure the minimum and maximum disparity thresholds (Dmin and Dmax) for both absolute and relative disparity. Consistent with previous studies, we found that Dmin thresholds are substantially worse for absolute disparity than for relative disparity. We tested three relative disparity mechanisms: (1) the difference between the monocular separations of targets projecting to the two eyes; (2) the direct measurement of relative disparity; and (3) the difference of absolute disparities of targets. Computing the difference of absolute disparities when detecting relative disparity, Mechanism 3 cancels global noise, resulting in a much lower relative Dmin threshold, and provides a reasonable fit to the experimental data. We also found that the presence of as much as 2400 arcsec of external disparity noise does not appear to affect the Dmax threshold. This observation suggests that Dmax is implicated in a mechanism that disregards the disparity variance of individual items, relying instead on the average disparity across all items, supporting the depth model proposed in our previous study (Ding & Levi, 2021), which posits distinct mechanisms governing Dmin and Dmax thresholds.

## Introduction

**Figure 1:**
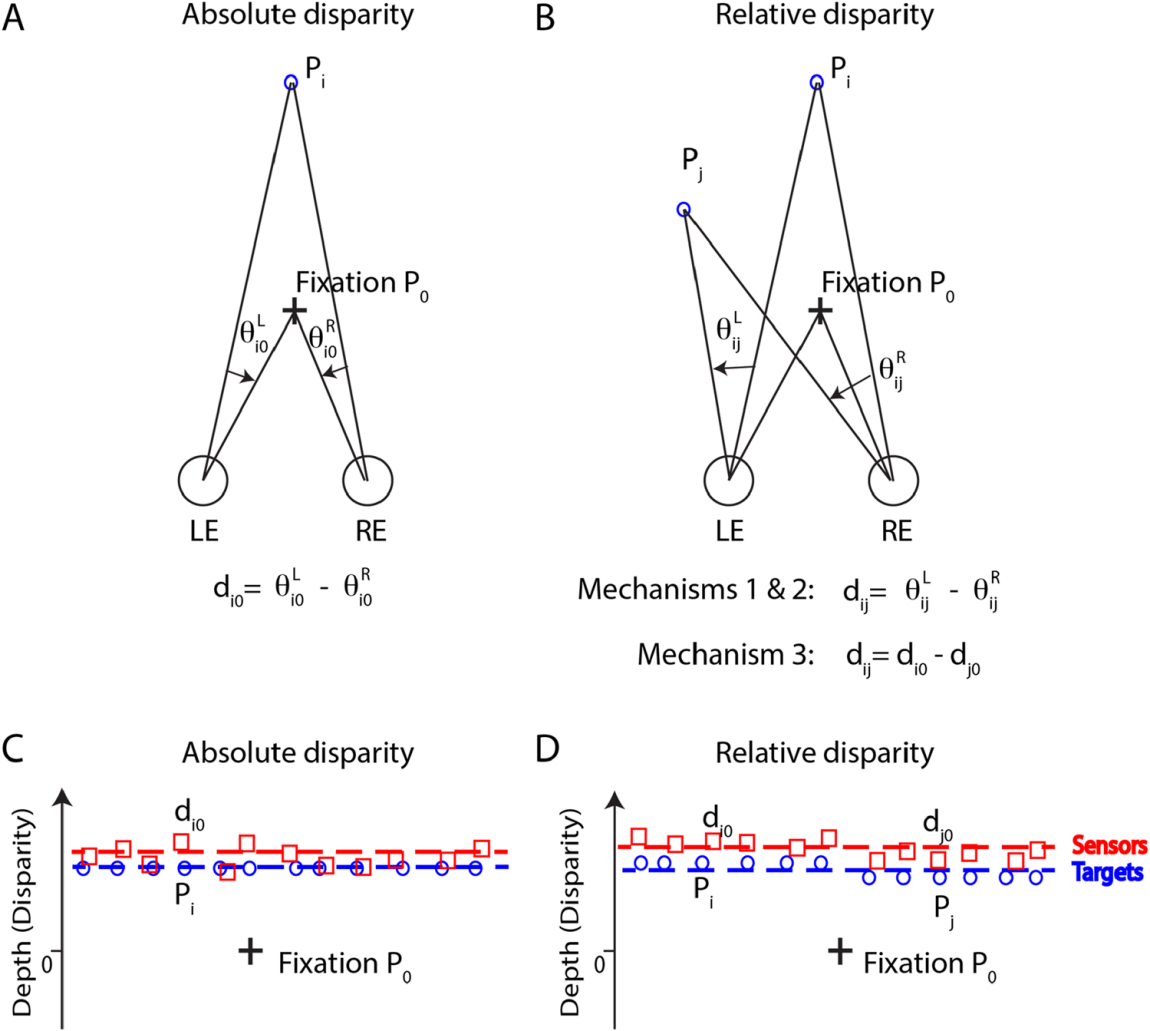
Schematic representations of absolute and relative disparity and internal disparity noise. (**A**) The absolute disparity of a point is defined as the difference of its visual angles related to the fixation point in the two eyes, i.e., the difference in the angular locations of its retinal images in the two eyes, referenced to corresponding retinal points (the disparity related to the fixation point). (**B**) Three mechanisms for calculating relative disparity between points P_*i*_ and P_*j*_: (1) the difference of the monocular separations ${\theta }_{ij}^{{\text{L}}}$ and ${\theta }_{ij}^{{\text{R}}}$ of targets projecting to the two eyes (Model 1); (2) the direct measurement of relative disparity ${d}_{ij}$ (Model 2); and (3) the difference of absolute disparities ${d}_{i0}$ and ${d}_{j0}$ of targets (Model 3). (**C**) A schematic representation of internal disparity noise for detecting absolute disparity. Although all targets (blue circles) have identical stimulus disparity with no variance, the disparity detectors’ absolute disparities vary from place to place, following a Gaussian distribution with variance ${\sigma }_{{\text{Loc}}}^{2}$ (Local variance). The mean disparity (red dashed line) of these detectors is still offset from the stimulus disparity (blue dashed line) and fluctuates from trial to trial, also following a Gaussian distribution with variance ${\sigma }_{{\text{Glob}}}^{2}$ (Global variance). Please note that the offset could vary on either side of the stimulus disparity. Assuming that the global and local noises are independent of each other, the total internal disparity variance is given by ${\sigma }_{{\text{Int}}}^{2}={\sigma }_{{\text{Loc}}}^{2}+{\sigma }_{{\text{Glob}}}^{2}$. (**D**) A schematic representation of internal disparity noise for detecting relative disparity (Mechanism 3). The targets in the two areas differ in stimulus disparity. The relative disparity *d*_*ij*_ between targets P_i_ and P_j_ located on the two areas can be measured as the difference of absolute disparities *d*_*i0*_ and *d*_*j0*_ of the two targets referenced to the fixation point P_0_. The global noise is canceled in the difference.

Stereopsis, the perception of depth through the brain's processing of binocular disparity, has been extensively studied^1–6^. The absolute disparity of a point in space is defined as the difference between the angle subtended by the target at the two entrance pupils of the eyes and the angle of convergence, i.e., the difference in the angular locations of the retinal images of the target in the two eyes, referenced to corresponding retinal points (the disparity related to the fixation point) (Fig. 1A). When a second point is located in a different depth plane, this introduces a relative disparity with the first point. However, there are three ways to calculate the relative disparity between two points (Fig. 1B): (1) the difference of the monocular separations of the two points projecting to the two eyes (Mechanism 1), i.e., the prior uniocular processing hypothesis^7^; (2) the direct measurement by a relative disparity mechanism (Mechanism 2); and (3) the difference of absolute disparities of the two points (Mechanism 3).

There are neurons in V1 that are tuned for absolute disparity and are insensitive to relative disparity. Their disparity tuning curves do not shift when the disparity of the surrounding area varies^8^. On the other hand there are also neurons in the brain areas downstream from V1 that are sensitive to relative disparity. Their disparity tuning curves shift depending on the surrounding disparity^9,10^. However, in a psychophysical study, it is difficult, or even impossible, to exclude the possibility that relative disparity information may be used for detecting absolute disparity if a fixation point or other background marks are visible^4^. Because of the absolute disparity anomaly^11^, the absence of conscious readout of absolute disparity, it is hard to perform the task of “pure” absolute disparity detection without a visible fixation point or other background references.

Here we propose three unified equivalent noise models, each combining one absolute disparity mechanism and one of three relative disparity mechanisms, to provide a unified explanation of two data sets of absolute and relative disparity minimum thresholds. To test the model we used stimuli with a fixation point for detecting absolute disparity to make the task less challenging, and we used stimuli without a fixation point for detecting relative disparity to test the possibility of relative mechanisms without prior absolute disparity processing. In general, we assume that both absolute and relative disparity mechanisms may be involved in performing either task. Our data and modeling support Mechanism 3, the difference of absolute disparities as the mechanism of measuring relative disparity. This is not surprising, given that spatial stereoresolution (relative disparity) is constrained by the calculation of absolute disparity through a binocular matching process^12^. Furthermore, hypercyclopean channels play a crucial role in the perception of disparity surfaces^13–16^.

### Absolute disparity

As shown in Fig. 1A, let ${\theta }_{i0}^{{\text{L}}}$ and ${\theta }_{i0}^{{\text{R}}}$ be the visual angles of point P_*i*_ referenced to the fixation point P_0_ in the two eyes respectively, i.e., monocular angular separations of point P_*i*_ from the fixation P_0_ in the two retinas. P_i_’s absolute disparity is given by:

1 $${d}_{i0}={\theta }_{i0}^{{\text{L}}}-{\theta }_{i0}^{{\text{R}}}.$$

In the physiological literature, there is clear evidence that both phase and position disparity sensitive neurons are present in cortical area V1^2,17,18^. Most likely, they are directly sensitive to absolute disparity without prior uniocular measurements of monocular angular separations ${\theta }_{i0}^{{\text{L}}}$ and ${\theta }_{i0}^{{\text{R}}}$ ^7,19^. Their absolute disparity tuning curves are not affected by the disparity in the surrounding area, i.e., they are insensitive to relative disparity^8^. These neurons may have different preferred disparities when detecting targets with the same stimulus disparity, i.e., there may be sampling errors when detecting absolute disparities (Fig. 1C). This internal disparity variance leads to inconsistent depth perception. Figure 1C provides a schematic representation of the disparity variance in absolute disparity detectors. In this figure blue circles represent targets with identical stimulus disparity, while red squares represent disparity detectors with varied preferred absolute disparities. We assume that the disparities of these detectors follow a Gaussian distribution with variance of ${\sigma }_{{\text{Loc}}}^{2}$ (local internal disparity noise). Their mean disparity (dashed red line), often offset from the stimulus disparity (dashed blue line), fluctuates from trial to trial because of resampling for a different trial by a different group of neurons, also following a Gaussian distribution with variance of ${\sigma }_{{\text{Glob}}}^{2}$ (global internal disparity noise). We further assume that the disparity response is proportional to the disparity with a random fluctuation that follows a Gaussian distribution. Therefore, the disparity response is given by:

2 $${R}_{i0}=A{d}_{i0}+N\left({\sigma }_{{\text{Int}}}^{2}\right).$$

where *A* is detection efficiency and $N\left({\sigma }_{{\text{Int}}}^{2}\right)$ is the internal disparity noise. If the disparity variance of one disparity detector is ${\sigma }_{{\text{Int}}}^{2}$, the variance of mean disparity of *M* detectors equals ${\sigma }_{{\text{Int}}}^{2}/M$. If the efficiency *A* = 1 for one disparity detector, the efficiency for *M* detectors would be $A=\sqrt{M}$. In other words, $M={A}^{2}$ disparity detectors are involved in disparity detection if the efficiency *A* completely reflects sampling efficiency.

We assume that the internal noise $N\left({\sigma }_{{\text{Int}}}^{2}\right)$ has both global and local components, i.e.,

3 $$N\left({\sigma }_{{\text{Int}}}^{2}\right)=N\left({\sigma }_{{\text{Glob}}}^{2}\right)+N\left({\sigma }_{{\text{Loc}}}^{2}\right).$$

Global noise $N\left({\sigma }_{{\text{Glob}}}^{2}\right)$ refers to random fluctuations in the visual system's response to binocular disparities across the entire visual field (e.g., offset of red and blue dashed lines in Fig. 1C), while local noise $N\left({\sigma }_{{\text{Loc}}}^{2}\right)$ pertains to the noise specific to individual disparity detectors (e.g., offsets of red squares from the red dashed line in Fig. 1C). Both global and local noises affect the absolute disparity threshold.

To enhance clarity, we have included a table in “Appendix C” summarizing symbols used in the text.

### Relative disparity mechanism 1

As shown in Fig. 1B, let ${\theta }_{ij}^{{\text{L}}}$ and ${\theta }_{ij}^{{\text{R}}}$ be the visual angles subtended by point P_*i*_ referenced to point P_*j*_ in the two eyes respectively, i.e., the monocular angular separations of point P_*i*_ from point P_*j*_ in the two retinas. P_*i*_’s relative disparity referenced to point P_*j*_ is given by:

4 $${d}_{ij}={\theta }_{ij}^{{\text{L}}}-{\theta }_{ij}^{{\text{R}}}.$$

We assume that the visual system first measures the monocular separations of the two targets in the two retinas, ${\theta }_{ij}^{{\text{L}}}$ and ${\theta }_{ij}^{{\text{R}}}$, and then performs the subtraction operation. If the responses to monocular separations are given by,

5 $${R}_{ij}^{{\text{L}}}=A{\theta }_{ij}^{{\text{L}}}+N\left({\sigma }_{{\text{Int}}}^{2}\right),\mathrm{ and }\,{R}_{ij}^{{\text{R}}}=A{\theta }_{ij}^{{\text{R}}}+N\left({\sigma }_{{\text{Int}}}^{2}\right)$$

and the internal position noise is independent in the two eyes, the relative disparity response is given by:

6 $${R}_{ij}={R}_{ij}^{{\text{L}}}- {R}_{ij}^{{\text{R}}}=A{\theta }_{ij}^{{\text{L}}}-A{\theta }_{ij}^{{\text{R}}}+N\left(2{\sigma }_{{\text{Int}}}^{2}\right)=A{d}_{ij}+N\left(2{\sigma }_{{\text{Int}}}^{2}\right).$$

We note that relative disparity mechanism 1, i.e., the prior uniocular processing hypothesis, was rejected experimentally by Berry^19^ and Westheimer and McKee^7^. In the present study, we performed modeling to see if Mechanism 1 is also rejected statistically.

### Relative disparity mechanism 2

If the visual system measures the relative disparity directly, which does not need prior measures of monocular angular separations ${\theta }_{ij}^{{\text{L}}}$ and ${\theta }_{ij}^{{\text{R}}}$, or prior measures of absolute disparities either, the disparity response is given by:

7 $${R}_{ij}=A{d}_{ij}+N\left({\sigma }_{{\text{Int}}}^{2}\right).$$

Here, we assume that the internal variance ${\sigma }_{{\text{Int}}}^{2}$ is equal when measuring monocular separation in Eq. 5 and binocular disparity in Eqs. (2) and (7). Due to the direct measurement of relative disparity in Mechanism 2 (Eq. 7), its internal variance is only half that of Mechanism 1 (Eq. 6), where relative disparity is calculated as the difference of the two monocular separations.

### Relative disparity mechanism 3

Using the fixation point P_0_ as the common reference, the visual system first measures the absolute disparities of P_*i*_ and P_*j*_, and then performs the subtraction operation on the two absolute disparities. The relative disparity of two points P_i_ and P_j_ is given by:

8 $${d}_{ij}={d}_{i0}-{d}_{j0}.$$

The two absolute disparities share a common reference (the fixation plane), allowing the cancellation of global noise—a random fluctuation across the entire visual field—in the difference calculation of Eq. (8). However, global noise cannot be canceled in relative disparity Mechanisms 1 and 2 due to the absence of a common reference point in these mechanisms. From Eq. (2), the disparity response is given by:

9 $${R}_{ij}={R}_{i0}- {R}_{j0}=A{d}_{i0}-A{d}_{j0}+N\left(2{\sigma }_{{\text{Loc}}}^{2}\right)=A{d}_{ij}+N\left(2{\sigma }_{{\text{Loc}}}^{2}\right).$$

Figure 1D provides a schematic representation of relative disparity Mechanism 3. For detecting the relative disparity *d*_*ij*_ between targets *i and j* located in two depth planes, the global noise (offset of red and blue dashed lines) is canceled by calculating the difference in their absolute disparities.

Although neurons tuned to relative disparities have been isolated^9,10^ and could be an independent system for relative disparity detection (Mechanism 2), they can also take their inputs from absolute disparity neurons (Mechanism 3) or from monocular neurons (Mechanism 1) to calculate relative disparities. In the present paper, we compare the three mechanisms statistically through modeling.

Although internal disparity noise plays a crucial role in limiting depth thresholds, directly measuring it can be challenging or even impossible. To estimate the equivalent internal noise, researchers often used an equivalent noise procedure^20,21^, e.g., introducing external disparity noise to the stimuli to estimate internal disparity noise^22–24^. Because of the absolute disparity anomaly^11^, the mechanism of absolute disparity is seldom directly addressed in the literature^4^. Previous research has primarily focused on understanding the thresholds for detecting relative disparity, often utilizing an equivalent noise model to describe the underlying mechanisms^22,23^. To avoid the absolute disparity anomaly, in the present study, we provided a visible fixation point for detecting absolute disparity, i.e., relative disparity information was also used for performing the task. However, using a unified model of absolute and relative disparities, we were able to reveal both the absolute and relative disparity mechanisms. Our data and modeling show that global internal noise is involved in detecting absolute disparity but is canceled in detecting relative disparity.

A portion of the present study has been published as an abstract^25^.

## Methods

**Figure 2:**
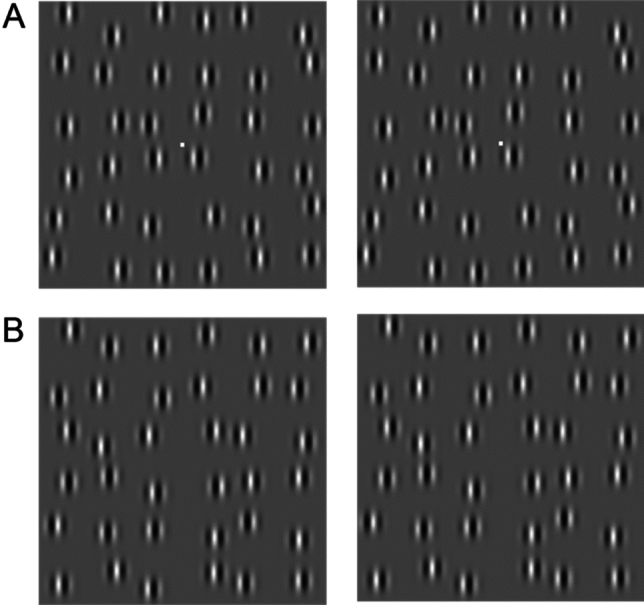
Random-Gabor-Patch (RGP) Stereograms. Gabor patches were positioned randomly and had random phases while maintaining a fixed spatial frequency. Both eyes were presented with identical arrays of patches, except for paired patches in the two eyes that could be randomly shifted in opposite directions following a Gaussian distribution, both horizontally and vertically. As a result, each pair of patches exhibited a random binocular disparity. The mean vertical disparity remained constant at zero, while the mean horizontal disparity had a non-zero value. (**A**) An array with either crossed or uncrossed mean horizontal disparity throughout the entire display. Absolute disparity thresholds were measured by presenting two intervals, one with crossed and the other with uncrossed stimulus disparity, with added external disparity noise $N\left({\sigma }_{{\text{Ext}}}^{2}\right)$. The observers’ task was to indicate which interval appeared closer. A fixation point is included. (**B**) The top and bottom regions of the array exhibit crossed or uncrossed mean horizontal disparity, respectively. Relative disparity thresholds were assessed using a single interval. The stimulus was divided into top and bottom halves, with either crossed or uncrossed mean horizontal disparity. The observers’ task was to indicate whether the top or bottom region of the array appeared closer. No fixation point is included.

*Stimuli.* Random-Gabor-Patch (RGP) stereograms (Fig. 2) were used as stimuli in this study. The stereograms consisted of vertical Gabor patches with random positions and phases, but a fixed spatial frequency. RGP stereograms provide stereoscopic depth signals within a narrow spatial frequency-and-orientation channel, eliminating monocular depth cues. Each eye was presented with an identical array of patches, except for paired patches that could undergo random shifts in opposite directions in the two eyes, following a Gaussian distribution both horizontally and vertically. This introduced a random binocular disparity to each patch pair, effectively acting as external disparity noise. The mean vertical disparity remained consistently at zero, while the mean horizontal disparity assumed a non-zero value. Figure 2A depicts an array with either crossed or uncrossed mean horizontal disparity spanning the entire display, thus serving as the stimulus for detecting absolute disparity. To minimize the impact of the absolute disparity anomaly, a fixation point was included. In Fig. 2B, the top and bottom halves of the array exhibit crossed and uncrossed mean horizontal disparity, respectively, serving as the stimulus for detecting relative disparity without a fixation point. It is important to note that the stimulus remained static during a single presentation of the stereogram. The jth Gabor patch pair is given by:

10 $${I}_{j{\text{L}}}={{m}_{{\text{L}}}e}^{-\frac{{\left(x-{x}_{j}-\frac{d}{2}-\frac{{n}_{jx}}{2}\right)}^{2}+{\left(y-{y}_{j}-\frac{{n}_{jy}}{2}\right)}^{2} }{2{\sigma }^{2}}}{\text{cos}}\left(\omega \left(x-{x}_{j}-\frac{d}{2}-\frac{{n}_{jx}}{2}\right)+{\theta }_{j}\right)$$

11 $${I}_{j{\text{R}}}={m}_{{\text{R}}}{e}^{-\frac{{\left(x-{x}_{j}+\frac{d}{2}+\frac{{n}_{jx}}{2}\right)}^{2}+{\left(y-{y}_{j}+\frac{{n}_{jy}}{2}\right)}^{2}}{2{\sigma }^{2}}}{\text{cos}}\left(\omega \left(x-{x}_{j}+\frac{d}{2}+\frac{{n}_{jx}}{2}\right)+{\theta }_{j}\right).$$

We tested five spatial frequencies [0.375, 0.75, 1.5, 3.0, and 6.0 cycles per degree (cpd)], with the number of patches being 4, 16, 36, 100, and 400 in a square measuring 14.1 × 14.1 degrees and the patch size in radius being 4.0, 2.0, 1.0, 0.5 and 0.25 degrees, respectively. The luminance contrast (*m*_L_, *m*_R_) of Gabor patches was always 100% in the two eyes. To create an RGP stereogram with 3.0 cpd Gabor patches, a large square measuring 14.1 × 14.1 degrees was divided into 10 × 10 small grids. Each grid contained a Gabor patch with a spatial frequency (ω) of 3 cpd and a standard deviation (σ) of 0.167 degrees, randomly distributed within the area (gridwidth – σ) x (gridwidth – σ) with equal distribution. The luminance profiles of a Gabor patch pair are given by Eqs. (10) and (11) when the patch radius is less than 4σ and were set to be zero otherwise. The visible size is approximately 3σ in radius, e.g., 0.5 degree for a patch with a spatial frequency of 3.0 cpd. Along the grid border, two patches could partially overlap. The local disparity of each paired patch was generated by shifting the patches by equal amounts (equal to half the local disparity) but in opposite directions. A circular shift was performed to maintain a constant stereogram size. The local disparity across the stereogram followed a Gaussian distribution. The mean vertical disparity was always zero, while the mean horizontal disparity was either a positive (uncrossed) or negative (crossed) value, representing the stimulus disparity (*d*). The standard deviation of local disparities represents the external noise. RGP stereograms with differently scaled Gabor patches were constructed using similar methods.

Absolute disparity thresholds were measured by presenting two intervals, one with crossed and the other with uncrossed stimulus disparity. Observers judged which interval appeared nearer. Each interval lasted for 1 s, with a 0.5-s inter-interval duration. Relative disparity thresholds were assessed using a single interval lasting for 1 s. The stimulus was divided into top and bottom halves, with either crossed or uncrossed mean horizontal disparity, and observers determined which half appeared closer.

Although a 2IFC (Two-Interval Forced Choice) task for measuring absolute disparity thresholds and a 2AFC (Two-Alternative Forced Choice) task for measuring relative disparity thresholds are two distinct experimental paradigms, they can both be analyzed using Signal Detection Theory (see the following Models section) to extract system internal noise and efficiency. Despite this common analytical approach, the two tasks differ in their memory demands, and a 2IFC task may exhibit asymmetry between the two intervals. To verify the comparability of results obtained from these two tasks, we conducted two control experiments using a 2IFC paradigm to measure relative disparity thresholds: (1) with a signal (relative disparity) plus noise in one interval and noise only in the other (2IFC-1), and (2) with a signal plus noise in both intervals, while altering the direction of relative disparity presented in the two intervals (2IFC-2). In 2IFC-1, the observer’s task was to identify which interval exhibited a relative disparity, whereas in 2IFC-2, the task was to identify which interval exhibited a relative disparity with the bottom half nearer. Our data and modeling indicate that the internal noise remains consistent in both the 2AFC and 2IFC tasks for relative disparity detection, with variations in their performance attributed to distinct efficiencies (Appendix B).

The method of constant stimuli was employed to measure the minimum and maximum disparity thresholds (Dmin and Dmax). Data for one spatial frequency channel was collected as a block of 1200 trials with 6 levels of external noise, 10 disparities, and 20 repeats. To effectively model the data depicting the probability of a correct response in relation to binocular disparity, we utilized a psychometric function based on the sum of two cumulative Gaussian distribution functions. One of these functions exhibited an increasing trend corresponding to Dmin, while the other displayed a declining trend for Dmax as the values of disparity increased. The specific values of Dmin and Dmax thresholds were determined as the disparities that produced a 75% correct response rate.

The stimuli were presented on a 22-inch NEC MultiSync CRT monitor with a spatial pixel resolution of 1920 × 1440 and a vertical refresh rate of 75 Hz. The experimental setup utilized a Linux System76 Mini running Matlab (MathWorks, Inc.) with the Psychophysics Toolbox extensions^26,27^. A specialized circuit^28^ was employed to achieve 14-bit gray-scale levels. Gamma correction was applied and verified by measuring 10 luminance levels using a Minolta LS-110 photometer. The minimum luminance of the monitor, with all pixels set to their lowest value, measured 0.2 cd/m^2^, while the maximum luminance, with all pixels set to their highest value, measured 74.2 cd/m^2^. The displays were viewed in a custom built 4 mirror stereoscope and positioned optically at 68 cm from the observer.

### Models

The following models are proposed to predict disparity minimum thresholds (Dmin) and not disparity maximum thresholds (Dmax).

#### Absolute disparity

Because the fixation point is fixed without external noise, when adding external noise $N\left({\sigma }_{{\text{Ext}}}^{2}\right)$ to the RGP stimuli, the system total noise equals $N\left({\sigma }_{{\text{Int}}}^{2}\right)+N\left({\sigma }_{{\text{Ext}}}^{2}\right)$. From Eq. (2), the response to the uncrossed disparity *d* is given by:

12 $${R}_{+}=Ad+N\left({\sigma }_{{\text{Int}}}^{2}\right)+N\left({\sigma }_{{\text{Ext}}}^{2}\right).$$

And the response to the crossed disparity *-d* is given by:

13 $${R}_{-}=-Ad+N\left({\sigma }_{{\text{Int}}}^{2}\right)+N\left({\sigma }_{{\text{Ext}}}^{2}\right).$$

A trial response is given based on

14 $${R}_{+}- {R}_{-}=2Ad+N\left(2{\sigma }_{{\text{Int}}}^{2}\right)+N\left(2{\sigma }_{{\text{Ext}}}^{2}\right).$$

Assuming that internal and external noises are independent, and that global and local internal noise are also independent, i.e., ${\sigma }_{{\text{Int}}}^{2}={\sigma }_{{\text{Loc}}}^{2}+{\sigma }_{{\text{Glob}}}^{2}$, the absolute minimum disparity threshold is given by:

15 $${D}_{{\text{min}}}^{{\text{Abs}}}=\frac{\sqrt{2{\sigma }_{{\text{Int}}}^{2}+2{\sigma }_{{\text{Ext}}}^{2}}}{2{A}_{{\text{Abs}}}}=\frac{\sqrt{2{\sigma }_{{\text{Loc}}}^{2}+2{\sigma }_{{\text{Glob}}}^{2}+2{\sigma }_{{\text{Ext}}}^{2}}}{2{A}_{{\text{Abs}}}}.$$

In the present study, absolute disparity thresholds were assessed using the 2IFC task, where the uncrossed disparity *d* was randomly assigned to either the first or second interval with an equal 50% probability. Any potential asymmetry between the two intervals can be mitigated in Eq. (14). This is achieved by formulating the equation based on ${R}_{1}- {R}_{2}$ for half of the trials where uncrossed disparity is in the first interval and ${R}_{2}- {R}_{1}$ for the remaining half of the trials where uncrossed disparity is in the second interval.

#### Relative disparity mechanism 1

Relative disparity thresholds were assessed using a single interval method of constant stimuli. The stimulus was divided into top and bottom halves, with either crossed or uncrossed mean horizontal disparity. Relative disparity between the top and bottom halves (${d}_{\pm }=2d$) is determined by the difference in monocular angular separations of a top point P_*i*_ from a bottom point P_*j*_ in the two retinas (Eq. 4)^7^. From Eq. (6), a trial response is given based on:

16 $${R}_{\pm }=2Ad+N\left(2{\sigma }_{{\text{Int}}}^{2}\right)+N\left(2{\sigma }_{{\text{Ext}}}^{2}\right)$$

The relative minimum disparity threshold based on Mechanism 1 (Eq. 16) is given by:

17 $${D}_{{\text{min}}}^{{\text{Rel}}}=\frac{\sqrt{2{\sigma }_{{\text{Int}}}^{2}+2{\sigma }_{{\text{Ext}}}^{2}}}{2{A}_{{\text{Rel}}}}$$

Please note that the global internal disparity noise cannot be canceled in Mechanism 1 (Eq. 16).

#### Relative disparity mechanism 2

Relative disparity between the top and bottom halves (${d}_{\pm }=2d$) is computed directly, without prior measurement of their monocular separations (Eq. 4) or prior measurement of their absolute disparities (Eq. 8). The global internal disparity noise cannot be canceled. From Eq. (7), a trial response is given based on:

18 $${R}_{\pm }=2Ad+N\left({\sigma }_{{\text{Int}}}^{2}\right)+N\left(2{\sigma }_{{\text{Ext}}}^{2}\right).$$

Because targets in both the top and bottom halves have independent external disparity noise $N\left({\sigma }_{{\text{Ext}}}^{2}\right)$, unlike the case of detecting absolute disparity in Eqs. (12) or (13), there is a total of $N\left(2{\sigma }_{{\text{Ext}}}^{2}\right)$ external noise when computing the relative disparity in Eq. (18). The relative minimum disparity threshold based on Mechanism 2 (Eq. 18) is given by:

19 $${D}_{{\text{min}}}^{{\text{Rel}}}=\frac{\sqrt{{\sigma }_{{\text{Int}}}^{2}+2{\sigma }_{{\text{Ext}}}^{2}}}{2{A}_{{\text{Rel}}}}.$$

#### Relative disparity mechanism 3

Relative disparity between the top and bottom halves is computed as the difference of their absolute disparities. Again, the responses to crossed and uncrossed disparities are given by Eqs. (12) and (13) respectively. Because the global noise is canceled in the difference of absolute disparities within a single interval, a trial response is given based on:

20 $${R}_{\pm }={R}_{+}- {R}_{-}=2Ad+N\left(2{\sigma }_{{\text{Loc}}}^{2}\right)+N\left(2{\sigma }_{{\text{Ext}}}^{2}\right).$$

The relative minimum disparity threshold is given by:

21 $${D}_{{\text{min}}}^{{\text{Rel}}}=\frac{\sqrt{2{\sigma }_{{\text{Loc}}}^{2}+2{\sigma }_{{\text{Ext}}}^{2}}}{2{A}_{{\text{Rel}}}}.$$

In combination with absolute disparity mechanism Eq. (15), we tested three relative disparity mechanisms (Eqs. 17, 19 and 21) by fitting the unified model to predict both absolute and relative minimum thresholds. Our data and modeling show that the unified model with relative disparity Mechanism 3 (Eqs. 15 and 21) provide the best fit to both data sets.

*Mechanism 3 for relative disparity in control experiments utilizing the 2IFC task.* In the 2IFC-1 task, one interval comprises both the signal (relative disparity) and noise, while the other contains only noise. The task is to indicate which interval contains the relative disparity signal. The response to the interval with both signal and noise is given by Eq. (20), i.e., ${R}_{\pm }=2Ad+N\left(2{\sigma }_{{\text{Loc}}}^{2}\right)+N\left(2{\sigma }_{{\text{Ext}}}^{2}\right)$ and the response to the interval only with noise is given by ${R}_{{\text{N}}}=N\left(2{\sigma }_{{\text{Loc}}}^{2}\right)+N\left(2{\sigma }_{{\text{Ext}}}^{2}\right)$. Therefore, a trial response is given based on:

22 $${R}_{\pm }- {R}_{{\text{N}}}=2Ad+N\left(4{\sigma }_{{\text{Loc}}}^{2}\right)+N\left(4{\sigma }_{{\text{Ext}}}^{2}\right).$$

The relative Dmin threshold is given by:

23 $${D}_{{\text{min}}}^{{\text{Rel}}}=\frac{\sqrt{4{\sigma }_{{\text{Loc}}}^{2}+4{\sigma }_{{\text{Ext}}}^{2}}}{2{A}_{{\text{Rel}}}}.$$

In the 2IFC-2 task, both intervals contain a signal plus noise, with the direction of relative disparity varying between the two intervals from trial to trial. The task is to indicate which interval exhibits a relative disparity with the bottom half nearer. Again, referring to Eq. (20), the response to one interval with signal plus noise is given by ${R}_{\pm }=2Ad+N\left(2{\sigma }_{{\text{Loc}}}^{2}\right)+N\left(2{\sigma }_{{\text{Ext}}}^{2}\right)$ and the response to the other interval with a reversed relative disparity plus noise is given by ${R}_{\mp }=-2Ad+N\left(2{\sigma }_{{\text{Loc}}}^{2}\right)+N\left(2{\sigma }_{{\text{Ext}}}^{2}\right)$. Therefore, a trial response is given based on:

24 $${R}_{\pm }- {R}_{\mp }=4Ad+N\left(4{\sigma }_{{\text{Loc}}}^{2}\right)+N\left(4{\sigma }_{{\text{Ext}}}^{2}\right).$$

The relative Dmin threshold is given by:

25 $${D}_{{\text{min}}}^{{\text{Rel}}}=\frac{\sqrt{4{\sigma }_{{\text{Loc}}}^{2}+4{\sigma }_{{\text{Ext}}}^{2}}}{4{A}_{{\text{Rel}}}}.$$

## Observers

Three observers with normal or corrected-to-normal vision signed an informed consent form and participated in the experiment. One observer is a coauthor, and the others are naïve observers. All observers were screened for stereoacuity better than 20 arcseconds using the clinical stereo circle test (Randot Stereotest, Stereo Optical Co., Inc.). The experimental protocol was approved by the internal board of the ethics committee (IRB) of University of California, Berkeley, according to the guidelines and regulations for human subject research. All experimental protocols were performed in accordance with the guidelines provided by the committee approving the experiments. The data were averaged across the three observers.

## Results

**Figure 3:**
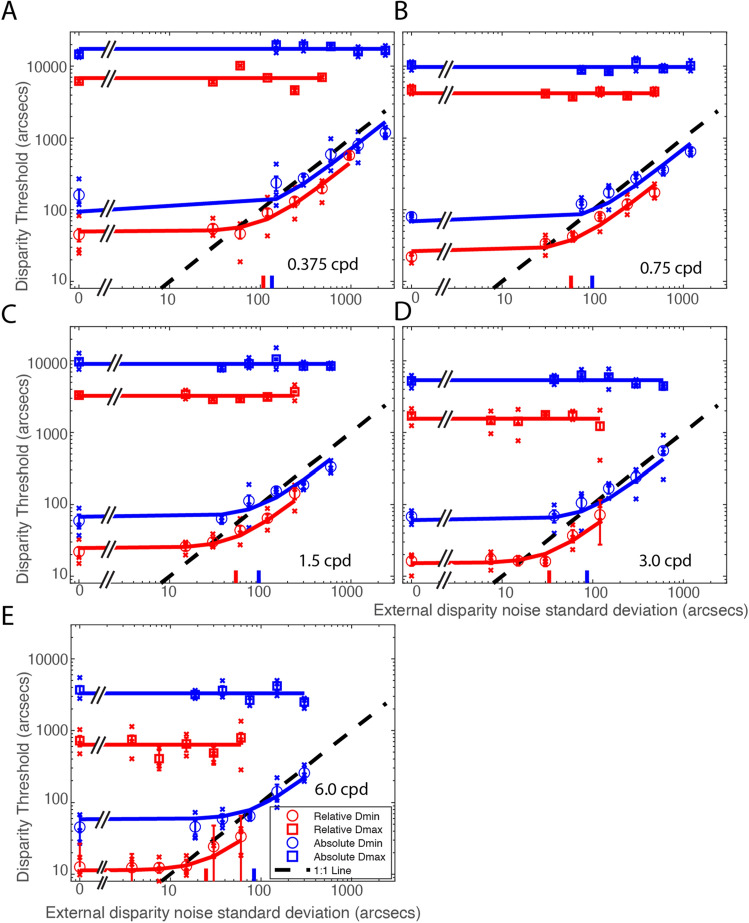
Results. Mean Disparity thresholds (Dmin and Dmax) of three observers as a function of external disparity noise standard deviation for detecting absolute disparity (Blue) or relative disparity (Red) at five spatial frequencies ranging from 0.375 to 6.0 cpd. The smooth curves for Dmin thresholds are the best fits of a unified equivalent noise model with both global and local internal disparity noise (Model 3: Eqs. 15 and 21). The horizontal lines indicate the average Dmax across all levels of external disparity noise. A slanted black dashed line (1:1 line) indicates the Dmin thresholds equal to external disparity noise standard deviation. The red and blue vertical bars on the x-axis indicate the standard deviations of equivalent internal disparity noise for relative and absolute disparity detections, respectively. Open circles and squares show the mean values across three observers, and x’s indicate individual observers’ data. The error bars represent standard errors.

Figure 3 shows Dmin and Dmax thresholds for detecting relative (Red) and absolute (Blue) disparities as a function of external disparity noise standard deviation. Dmax threshold remains constant (the average is indicated by horizontal lines in Fig. 3, not predictions from a model) independent of external noise for both relative and absolute disparities, indicating that the disparity variance may have no effect on the upper disparity limit under conditions of the present study (maximum external noise standard deviation ≈ 960 arc sec for relative or 2400 arc sec for absolute disparity detection). On the other hand, for both tasks, Dmin threshold remains constant at small external noise levels but increases proportionally with external noise at large noise standard deviations, with the turning point (indicated by a color vertical bar on x-axis in Fig. 3) estimated as the standard deviation of equivalent internal disparity noise. The smooth colored curves for Dmin thresholds are the best fits of Model 3 (Eqs. 15 and 21), a unified equivalent noise model with both global and local internal disparity noise. Dmin threshold is lower in relative than in absolute disparity detection at all external noise levels. Model 3 provides a unified account of both absolute and relative Dmin thresholds.

**Figure 4:**
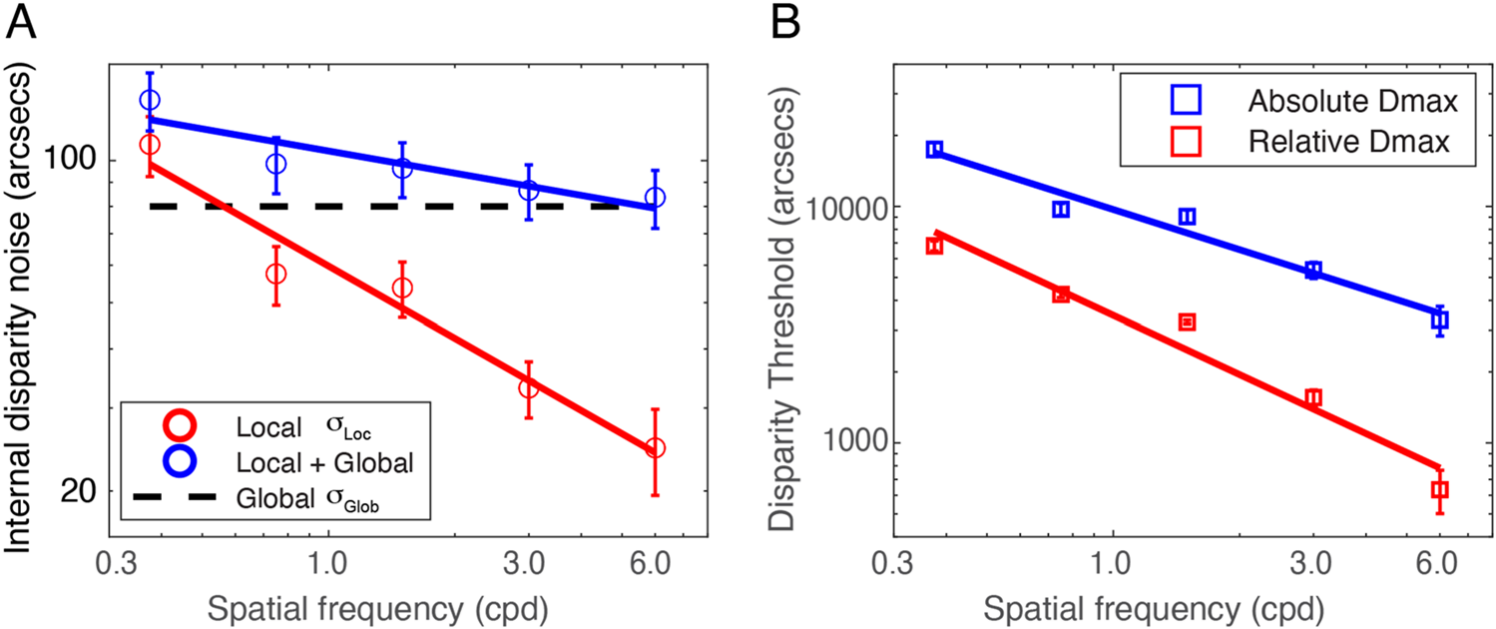
(**A**) The standard deviation of internal disparity noise, ${\sigma }_{{\text{Loc}}}$, $\sqrt{{\sigma }_{{\text{Loc}}}^{2}+{\sigma }_{{\text{Glob}}}^{2}}$, and ${\sigma }_{{\text{Glob}}}$, as a function of spatial frequency. (**B**) Absolute (Blue) and relative (Red) Dmax as a function of spatial frequency.

Our modeling shows that detecting relative disparity is more efficient and has lower internal noise than detecting absolute disparity. Model 3 (Eqs. 15 and 21) has eight parameters, one global noise standard deviation (${\sigma }_{{\text{Glob}}}$) for absolute disparity detection (but canceled in relative disparity detection), two equivalent efficiencies (${A}_{{\text{Abs}}}$ and ${A}_{{\text{Rel}}}$) for detecting absolute and relative disparities respectively and five local noise standard deviations (${\sigma }_{{\text{Loc}}}$) corresponding to five different scales for both absolute and relative disparity detection. The equivalent efficiency, ${A}_{{\text{Abs}}}=$ 1.01 ± 0.06 (~ 1 detector) for detecting absolute disparity and ${A}_{{\text{Rel}}}=$ 1.54 ± 0.10 (~ 2 detectors) for detecting relative disparity, is independent of spatial frequency. This is consistent with Wardle, Bex et al.^23^, who reported that the efficiency for depth discrimination (relative disparity) was consistently very low (1–4 detectors) across the visual field. However, as far as we know, the efficiency for detecting absolute disparity had not been measured before. We speculate that the higher efficiency for detecting relative (compared to absolute) disparity in the present study may be due to having more disparity detectors involved in detecting relative disparity. Lower internal noise in detecting relative (compared to absolute) disparity may be because the global internal disparity noise, which is involved in detecting absolute disparity, can be canceled out in the detection of relative disparity. As shown in Fig. 4A, the local internal disparity standard deviation ${\sigma }_{{\text{Loc}}}$, which may be partially caused by sampling errors, decreases as spatial frequency increases (slope ≈ − 0.51 in log–log coordinates). In contrast, the global internal disparity ${\sigma }_{{\text{Glob}}}$ (= 79.9 ± 10.6) appears to be independent of spatial frequency, resulting in a slower decrease of the combined global and local internal disparity noise (total internal noise for detecting absolute disparity: $\sqrt{{\sigma }_{{\text{Loc}}}^{2}+{\sigma }_{{\text{Glob}}}^{2}}$) as spatial frequency increases (slope ≈ − 0.16 in a log–log plot). This explains why the Dmin threshold decreases more rapidly for detecting relative than absolute disparities when spatial frequency increases (Fig. 3).

We initially performed a preliminary experiment using a spatial frequency of 1.5 cpd to estimate an appropriate range of test noise levels. The estimation was based on the assumption that the internal disparity noise decreases with a log–log slope of − 1 as spatial frequency increases, aiming to maintain constancy in phase disparity space. However, our findings indicate that the reduction in internal disparity noise is much slower than initially anticipated with increasing spatial frequency, as illustrated in Fig. 4A. Consequently, we performed a limited number of tests with lower noise levels at lower spatial frequencies, resulting in suboptimal fits of the horizontal lines in Fig. 3. These fits are particularly influenced by the threshold in the absence of external noise.

Both relative and absolute Dmax thresholds decrease as spatial frequency increases (Fig. 4B). However, the relative disparity Dmax decreases more rapidly than the absolute disparity Dmax (with a slope of approximately − 0.83 compared to − 0.57 in log–log coordinates).

## Modeling

We used the Akaike Information Criterion (AIC), a measure of the relative goodness of fit of a statistical model developed by Akaike^29^, to compare different models (See Appendix A).

Table 1 shows chi square values and AICc scores for model fitting and statistical comparisons of three unified models, each combining one absolute (Eq. 15) and one of three relative disparity mechanisms (Eqs. 17, 19 and 21). The ‘best’ model is the one with the lowest AICc score. The Akaike weight (Aw), the relative likelihood of a model being the ‘best’ one in the set of models being considered, is given in the last column. Model 3 is the best, with a 93.08% Akaike weight. Model 1 (Eqs. 15 and 17) has 7 model parameters, two efficiencies (${A}_{{\text{Abs}}}$ and ${A}_{{\text{Rel}}}$) for detecting absolute and relative disparities respectively and five deviations (${\sigma }_{{\text{Int}}}$) of internal noise corresponding to five different scales for both absolute and relative disparity detection. The threshold difference for detecting absolute and relative disparities is accounted for only by their different detection efficiencies. However, greater efficiency for detecting relative disparity is not sufficient to explain all the data sets. It’s likelihood for the ‘best’ model (Aw) is very low, less than 0.01%. Model 2 (Eqs. 15 and 19) is almost identical to Model 1, except that the internal variance for detecting relative disparity is only a half of that of Model 1, because the relative disparity is measured directly in Model 2 while it is measured in two stages in Model 1. The lower internal variance for detecting relative disparity in Model 2 is helpful in accounting for the lower relative disparity threshold, increasing the likelihood to 6.91%. However, the constant 50% decrease in internal variance for detecting relative disparity fails to account for the observed fact that the decrease of internal variance for detecting relative disparity is scale dependent. As shown in Fig. 3, when spatial frequency increases, the internal noise decreases more for relative disparity than for absolute disparity detection. Adding a new parameter of Global noise standard deviation (${\sigma }_{{\text{Glob}}}$), Model 3 (Eqs. 15 and 21) has eight parameters. Because the global noise is canceled in detecting relative disparity in the difference of absolute disparities in Model 3, the threshold is lower for relative than for absolute disparity. Because the global noise is independent of scale (dashed black line in Fig. 4A), Model 3 successfully predicts that the internal noise is decreased more quickly in relative than in absolute disparities as spatial frequency increases (blue and red lines in Fig. 4A). Its likelihood to be the best model is 93.08%.

**Table 1:** Fitting statistics of unified models with three different relative mechanisms.

	K	$\upnu$	${\upchi }^{2}$	${\upchi }^{2}/\upnu$	AICc	Aw
Model 1	7	54	129.4	2.40	64.6	< 0.01%
Model 2	7	54	97.9	1.81	47.6	6.91%
Model 3	8	53	85.9	1.62	42.4	93.08%

K, the number of model parameters; $\upnu$, the number of degrees of freedom; AICc, Akaike Information Criterion with a correction; Aw, Akaike weight.

## Discussion

Although previous studies have tried to study the absolute disparity mechanism based on “pure” absolute disparity detection tasks^4,11^ performed without any visible marks as a reference in the background, such “pure” absolute disparity tasks may not exist in natural viewing conditions. In our daily life, the depth of a target is always perceived with visible surrounding references. Even in a lab environment, where any visible marks may be removed from the background, an invisible reference from memory may still be needed to accurately perceive depth^4,11^. In fact, any depth perception needs both absolute and relative disparity mechanisms, whether detecting absolute or relative disparities. This makes it difficult, or even impossible, to isolate each mechanism with psychophysical experiments. Although neurons tuned to absolute^8^ and relative^9,10^ disparities have been isolated, the debate over the relative disparity mechanism has never been completely settled^4,11,30^. The present study is an effort to study the two mechanisms in combination, using a new model of the two mechanisms, which provides a unified explanation of both absolute and relative detection tasks in a comprehensive framework.

Our data and modeling show that detecting relative disparity requires prior absolute disparity processing, even when no fixation point is present when performing a relative disparity task, which aligns with findings from previous studies^7,11–16,31–33^. Tyler^13^ reported stereoscopic tilt and size aftereffects and suggested that visual processing at the hypercyclopean level involves feature-selective channels tuned for both size and orientation of stimulus elements. Subsequently, these hypercyclopean channels were employed to explain anisotropies in stereoacuity for disparity corrugations^15,31–33^. The present study suggests that the global internal disparity noise is effectively canceled through the relative disparity calculation within these hypercyclopean channels.

**Figure 5:**
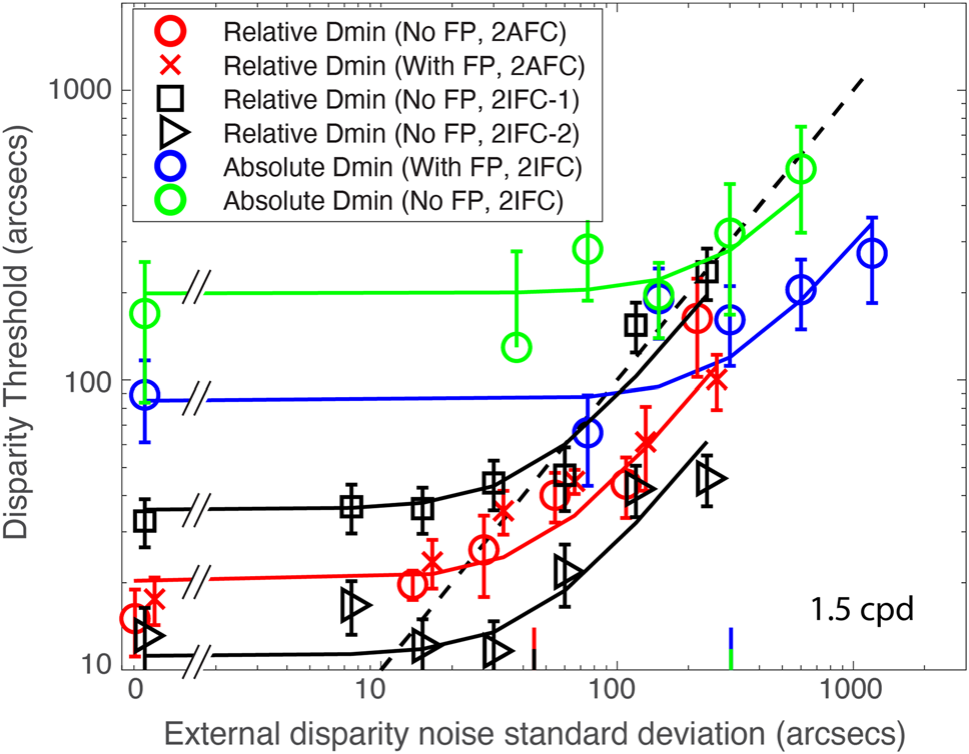
The comparison of absolute disparity thresholds with (With FP—blue circles) or without (No FP—green circles) a fixation point, the comparison of relative disparity thresholds with (With FP—red x’s) or without (No FP—red circles) a fixation point, and the comparison of relative disparity thresholds measured via either 2AFC (red x’s and circles) or 2IFC (black squares and triangles) tasks. The data was collected from one of the three participants in Fig. 3. The smooth curves are the best fits of a unified equivalent noise model with one global and one local internal disparity noises (Model 3). A single red curve fits both data sets (with a small horizontal offset in x-axes for better data presentation) of relative disparity thresholds collected from 2AFC task with and without a fixation point. A slanted black dashed line (1:1 line) indicates the Dmin thresholds equal to external disparity noise standard deviations. The red and black vertical bars (overlapping each other) on the x-axis indicate the standard deviation of equivalent internal disparity noise for relative disparity detections, measured via 2AFC (either with or without a fixation point presented) and 2IFC tasks respectively. The blue and green vertical bars (overlapping each other) on x-axis indicate the standard deviations of equivalent internal disparity noise for absolute disparity detections, with or without a fixation point respectively. The error bars represent standard errors.

As a comparison to the present results, we performed a control experiment for detecting relative disparity with a fixation point (Red x’s in Fig. 5) and found no difference whether a visible fixation point was present (Red x’s in Fig. 5) or not (Red circles in Fig. 5). On the other hand, relative disparity information may also be necessary to reveal the absolute disparity mechanism. Providing a visible fixation point as a reference is helpful in detecting absolute disparity, minimizing the impact of the absolute disparity anomaly^11^. Our participants showed much worse performance for detecting absolute disparity without a fixation point.

Figure 5 shows the comparison of two absolute disparity Dmin data sets—one with a fixation point (Blue circles) and one without (Green circles), both collected from the 2IFC task. It also presents four relative Dmin datasets under different conditions: (1) with a fixation point, collected from the 2AFC task (Red x’s); (2) without a fixation point, collected from the 2AFC task (Red circles); (3) without a fixation point, collected from the 2IFC-1 task with a signal (relative disparity) plus noise in one interval and noise only in the other (Black squares); and (4) without a fixation point, collected from the 2IFC-2 task featuring a signal plus noise in both intervals but with a reversal in the direction of relative disparity presented in the two intervals (Black triangles). The data were collected from one of the three participants in Fig. 3. The smooth curves are the best fits to the six data sets of a unified equivalent noise model (Model 3) with seven parameters as shown in Table 2: global and local noise standard deviations and five detection efficiencies. The standard deviations of equivalent internal disparity noise are indicated by colored vertical bars on the x-axis in Fig. 5. Please note that a single curve (Red) fits both relative Dmin data sets collected from the 2AFC task with (Red x’s) and without (Red circles) a fixation point.

**Table 2:** Model parameters for fitting control experiments (Fig. 5).

	${\sigma }_{{\text{Glob}}}$	${\sigma }_{{\text{Loc}}}$	${A}_{{\text{Abs}}}^{{\text{NoFP}}}$	${A}_{{\text{Abs}}}^{{\text{FP}}}$	${A}_{{\text{Rel}}}^{2{\text{AFC}}}$	${A}_{{\text{Rel}}}^{2{\text{IFC}}-1}$	${A}_{{\text{Rel}}}^{2{\text{IFC}}-2}$
Model 3	300.3 ± 90.3	42.3 ± 5.5	1.08 ± 0.30	2.52 ± 0.52	1.45 ± 0.17	1.20 ± 0.14	1.95 ± 0.20

${\sigma }_{{\text{Glob}}}^{2}$, ${\sigma }_{{\text{Loc}}}^{2}$ : global and local internal disparity variance, respectively.

${A}_{{\text{Abs}}}^{{\text{FP}}}$, ${A}_{{\text{Abs}}}^{{\text{NoFP}}}$: detection efficiency of absolute disparity with and without a fixation point, respectively.

${A}_{{\text{Rel}}}^{2{\text{AFC}}}$, ${A}_{{\text{Rel}}}^{2{\text{IFC}}-1}$, ${A}_{{\text{Rel}}}^{2{\text{IFC}}-2}$: detection efficiency of relative disparity using 2AFC, 2IFC-1 and 2IFC-2 tasks, respectively.

Our modeling indicates that incorporating a fixation point is more likely (73.4%) to enhance the efficiency of absolute disparity detection, rather than reducing internal noise (22.5%) (see Appendix B). Assuming that global noise cancellation occurs only within the same spatial-frequency band, the internal noise may remain unchanged in absolute disparity detection, regardless of the presence of a fixation point (as depicted in Fig. 5). This is because the disparity targets are confined to a narrow-banded channel, while the fixation point spans a broader band. The addition of a fixation point reduces the absolute disparity anomaly^11^, thereby increasing the efficiency of absolute disparity detection. However, given the limited conditions examined in our control experiment, we cannot conclusively rule out the possibility that a fixation point might effectively reduce global internal noise, potentially mitigating the absolute disparity anomaly. We leave further exploration of this topic to future studies. Conversely, the addition of a fixation point has no impact on relative disparity detection. The internal noise in relative disparity detection remains consistent in both the 2AFC and 2IFC tasks, with their differing performance attributed to distinct efficiencies (see Appendix B). Importantly, the global internal noise can be effectively canceled in relative disparity detection using either the 2AFC or 2IFC task.

On the other hand, by canceling global disparity noise, observers are highly sensitive to relative disparity even during eye/head movements and are essentially blind to large changes in absolute disparity^34–36^. Indeed, Erkelens and Collewijn^35,36^ reported that relative depth perception is independent of vergence errors. Steinman, Collewijn and co-workers^37,38^ concluded that relative horizontal disparity alone determines stereo thresholds and that a shift in vergence posture, which alters the absolute retinal disparities across the entire visual field, does not degrade stereopsis.

However, Ukwade, Bedell and Harwerth^39^ found that the stereo threshold is elevated if the vergence error exceeds a critical value (around 90 arcseconds) regardless of whether the vergence error was induced by forced vergence or was simulated by disconjugate retinal image motion. They explained this increase in stereo threshold by comparing the induced vergence error to a disparity pedestal. Indeed, numerous studies have reported that stereoacuity thresholds rise exponentially as the pedestal disparity increases^40–45^. We speculate that under natural viewing conditions, vergence errors and possibly other forms of internal global disparity noise have evolved to be well-suited for stereo vision, minimizing their impact on stereoacuity.

Considering the induced vergence error in Ukwade, Bedell and Harwerth^39^ as external global disparity noise, when it exceeds a critical value (around 90 arcseconds), i.e., the internal global disparity noise, the stereo threshold is elevated. The internal global disparity noise measured in the present study is approximately 80 arcseconds, which aligns closely with their findings of around 90 arcseconds^39^. While vergence noise may constitute a significant portion of global disparity noise, uncorrelated motor noise in the two eyes during eye/head movements may also contribute to this global noise. Furthermore, in addition to these sources of global noise, neuron resampling, which results in varying mean disparities among different neuron groups, may also contribute to the overall global noise. All these forms of global noise are relevant to the normal constraints on human vision.

In the present study the acuity for relative disparity was, on average, approximately 3.5 times better than that for absolute disparity with a fixation point presented. This result is consistent with Chopin et al.'s 2016 study^11^, which reported an average improvement of ≈ 4 times for relative disparity compared with “pure” absolute disparity thresholds measured by attempting to exclude any visible reference marks. However, without a visible fixation point, our participants showed much worse performance for detecting absolute disparity than Chopin’s. Compared with relative disparity detection, one of our participants showed about 10 times worse performance (Fig. 5) and another participant was unable to provide consistent data. We speculate that Chopin’s participants may have been well trained to use a reference from memory when detecting absolute disparity without a visible reference.

Our findings indicate that the Dmax threshold is influenced by spatial frequency (Fig. 4B), aligning with the results of a previous study^46^. However, it's important to note that in our experiments, spatial frequency and size were correlated, which raises the possibility that our results may reflect a dependency on stimulus size^47^. Under the conditions of the present study, adding external disparity noise (at least up to a standard deviation of ≈ 2400 arc sec for absolute or 960 arc sec for relative disparity) does not appear to affect the Dmax threshold (Fig. 3), which suggests that Dmax may involve a mechanism that ignores local disparity variance but relies on the mean disparity of all items. This is consistent with a previous study showing that the Dmax threshold was determined by a non-linear coarse stereopsis mechanism, such as a second-order process^48^ or envelope extraction^47^, which disregards the local differences of individual targets^49,50^. Conversely, Dmin threshold depends on the local details of individual targets. Just 1 or 2 items may be sampled for disparity detection at the Dmin threshold level during a trial if the equivalent efficiency (1.01 for absolute and 1.54 for relative disparity detections) mainly reflects sampling efficiency, i.e., most of the disparity samples are neglected in the performance of the task^24^. This is consistent with previous studies^23,24^, which suggest that disparity signals are not globally integrated to extract the mean depth, and supports the model proposed by Ding and Levi^48^, which suggests that Dmin and Dmax are regulated by two distinct mechanisms.

In the present study, we proposed a novel unified equivalent noise model that incorporates both global and local internal disparity noise to provide a comprehensive framework for both absolute and relative disparities. Our model offers a crucial insight into the detection of absolute and relative disparity. Both global and local internal disparity noise affect detecting absolute disparity. However, for detecting relative disparity, which involves comparing differences between absolute disparities, our modeling shows that the influence of global noise on absolute disparity cancels out, leading to a lower threshold for detecting relative disparity compared to absolute disparity. Indeed, the dynamics^51^ and developmental trajectories^52,53^ of absolute and relative disparity are different. Moreover our conclusion that distinct mechanisms govern Dmin and Dmax thresholds is consistent with the finding that coarse stereopsis develops earlier^54^ and is less susceptible to the effects of abnormal visual experience^55^, than fine stereopsis.

The establishment of this unified model contributes to a deeper understanding of the underlying mechanisms of absolute and relative disparities involved in stereovision. Furthermore, the findings from our study have the potential to address depth deficits in abnormal binocular vision, offering valuable insights that can aid in the diagnosis and treatment of such conditions.

### Supplementary Information

Supplementary Information.

## Data Availability

The datasets generated during and/or analyzed during the current study are available from the corresponding author, Jian Ding jian.ding@berkeley.edu, on reasonable request.
