# Supplementary material for: Absolute and relative disparity mechanisms revealed by an equivalent noise analysis
Source: Sci Rep. 2024 Mar 22;14:6863. doi: 10.1038/s41598-024-57406-2 (PMC10958039; doi:10.1038/s41598-024-57406-2)
Supplement: Supplementary file 1 — Supplementary Information. [file 41598_2024_57406_MOESM1_ESM.docx]

**Appendix A.** Analysis of Akaike Information Criterion (AIC)

Let K be the number of estimated parameters in the model and $L_{\mathrm{Max}}$ be the maximized value of the likelihood function for the model, AIC is defined as $AIC=2K-2\ln L_{\mathrm{Max}}$. Assuming that the errors are normally distributed and independent, after ignoring the constant term, AIC is given by

$AIC=Nln\left( \frac{\chi^{2}}{N} \right)+2K$ , (A1)

where $\chi^{2}$ is the residual sum of square in the least squares fitting and N is the number of observed data points. To give a greater penalty for additional parameters, Burnham and Anderson^1^ recommended the AIC with a correction for finite sample sizes (AICc), which is given by,

$AICc=AIC+\frac{2K\left( K+1 \right)}{N-K-1}$ . (A2)

For the set of *M* models, the one with the lowest AICc score is most likely to be the best model of those considered. The relative likelihood of model *i* is proportional to $exp(-0.5\Delta_{i})$, where $\Delta_{i}$ is the AICc difference between model *i* and the best model (with the lowest AICc). Given the data and the set of *M* models, the relative likelihood or Akaike weight is given by (Burnham and Anderson, 2002):

$w_{i}=\frac{exp(-0.5\Delta_{i})}{\sum_{m=1}^{M} exp(-0.5\Delta_{m})}$ . (A3)

The AIC allows one to decide which model, of those considered in the analysis, is most likely to be the 'best' one - meaning closest in information-theoretic terms to an unknown 'true' model that is not (and could not be) in the set of models considered. Putting it another way, if none of the models is any good, picking the model with the lowest AICc will not identify a good model, and certainly not a 'correct' one, just the least-worst model. In summary, the chosen model is the one that minimizes the Kullback-Leibler distance between the model and the truth over the set of models considered^1^.

**Appendix B.** Modeling the control experiments (Figure 5)

*Impact of introducing a fixation point on absolute Dmin thresholds*

The inclusion of a fixation point had a profound impact on enhancing the performance in detecting absolute Dmin thresholds, as illustrated in Figure 5 (blue and green circles). This improvement can be attributed to increased efficiency and/or reduced internal noise: (1) The inclusion of a fixation point increases efficiency without influencing internal noise (Abs-M1 model); (2) The inclusion of a fixation point reduces internal noise without affecting efficiency (Abs-M2 model); (3) The inclusion of a fixation point leads to both increased efficiency and decreased internal noise (Abs-M3 model). The Abs-M1 model comprises three parameters, including one internal noise standard deviation shared by two datasets and two efficiencies for two datasets. In contrast, the Abs-M2 model comprises three parameters, including two internal noise standard deviations for two datasets and one efficiency shared by two datasets. The Abs-M3 model (the full model) features four parameters, with one internal noise standard deviation and one efficiency for each of the two datasets.

Table A1 presents chi-square values and AICc scores for model fitting, along with statistical comparisons of the three Abs-M1-3 models for absolute Dmin thresholds with and without a fixation point. The optimal model is determined by the lowest AICc score. The Akaike weight (Aw), representing the relative likelihood of a model being the 'best' within the set, is presented in the last column. Notably, the Abs-M1 model, assuming increased efficiency with the addition of a fixation point, emerges as the best with a 73.4% Akaike weight. However, it is worth noting that the Abs-M2 model, assuming decreased internal noise with the addition of a fixation point, still holds a 22.5% probability of being the correct model.

Table A1: Fitting statistics of models for absolute Dmin thresholds

|  | K | $\nu$ | $\chi^{2}$ | $\chi^{2}/\nu$ | AICc | Aw |
| --- | --- | --- | --- | --- | --- | --- |
| Abs-M1 | 3 | 9 | 7.29 | 0.81 | 7.73 | 73.4% |
| Abs-M2 | 3 | 9 | 8.90 | 0.99 | 10.1 | 22.5% |
| Abs-M3 | 4 | 8 | 6.98 | 0.87 | 13.5 | 4.1% |

K: the number of model parameters; $\nu$: the number of degrees of freedom;

AICc: Akaike Information Criterion with a correction; Aw: Akaike weight.

*Comparation of 2AFC and 2IFC tasks for relative Dmin threshold detection*

On the other side, the incorporation of a fixation point showed no discernible impact on the performance of detecting relative Dmin thresholds, as demonstrated in Figure 5 (red circles and x’s). However, 2AFC performance may be different from 2IFC performance. Here we compared data collected using the 2AFC task (red circles and x’s in Figure 5) with data obtained from the 2IFC task, where the relative disparity signal is present in one of two intervals (2IFC-1, black squares in Figure 5) or in both intervals (2IFC-2, black triangles in Figure 5) with a reversal of relative disparity directions in the two intervals. Three models were tested: (1) The two different tasks have no effect on internal noise but influences efficiency (Rel-M1 model); (2) The two different tasks affect internal noise without impacting efficiency (Rel-M2 model); (3) The two different tasks affects both internal noise and efficiency (Rel-M3 model). The Rel-M1 model consists of five parameters, including one internal noise standard deviation shared by four datasets and four efficiencies for four datasets. In contrast, the Rel-M2 model comprises five parameters, incorporating four internal noise standard deviations for four datasets and one shared efficiency for four datasets. The Rel-M3 model (the full model) features eight parameters, encompassing one internal noise standard deviation and one efficiency for each of the four datasets.

Table A2 presents chi-square values and AICc scores for model fitting, along with statistical comparisons of the three Rel-M1-3 models for relative Dmin thresholds collected from 2AFC with fixation point, 2AFC without fixation point, 2IFC-1, and 2IFC-2 tasks. Notably, the Rel-M1 model, assuming constant internal noise for datasets collected from different tasks, emerges as the best with an 98.3% Akaike weight. Conversely, the Rel-M2 model, assuming varied internal noise for different tasks, has a very low probability (0.5%) of being the correct model. Based on reduced chi-square values ($\chi^{2}/\nu$), model fitting didn’t improve by adding three more parameters to update Rel-M1 to the full model Rel-M3, which holds only a 1.2% probability of being correct.

Table A2: Fitting statistics of models for relative Dmin thresholds

|  | K | $\nu$ | $\chi^{2}$ | $\chi^{2}/\nu$ | AICc | Aw |
| --- | --- | --- | --- | --- | --- | --- |
| Rel-M1 | 5 | 21 | 24.9 | 1.18 | 15.3 | 98.3% |
| Rel-M2 | 5 | 21 | 37.3 | 1.77 | 25.8 | 0.5% |
| Rel-M3 | 8 | 18 | 21.4 | 1.19 | 24.2 | 1.2% |

K: the number of model parameters; $\nu$: the number of degrees of freedom;

AICc: Akaike Information Criterion with a correction; Aw: Akaike weight.

**Appendix C.** Symbols Table

P_0_ the fixation point

P*_i_* _,_ P*_j_* the ith and jth points in the visual field

$\theta_{i0}^{L}$, $\theta_{i0}^{R}$ monocular angular separations of point P*_i_* from the fixation P_0_ in the two retinas

$d_{i0}$ the ith point P_i_’s absolute disparity

$d_{j0}$ the jth point P_j_’s absolute disparity

$R_{i0}$ the response to absolute disparity $d_{i0}$

$R_{j0}$ the response to absolute disparity $d_{j0}$

$d_{ij}$ P*_i_*’s relative disparity referenced to point P*_j_*

$R_{ij}$ the response to relative disparity $d_{ij}$

$\theta_{ij}^{L}$ , $\theta_{ij}^{R}$ the monocular angular separations of point P*_i_* from point P*_j_* in the two retinas

$R_{ij}^{L}$, $R_{ij}^{R}$ the responses to monocular separations $\theta_{ij}^{L}$ and $\theta_{ij}^{R}$

*A* detection efficiency

$A_{\mathrm{Abs}}$ detection efficiency of absolute disparity

$A_{\mathrm{Rel}}$ detection efficiency of relative disparity

$\sigma_{\mathrm{Loc}}^{2}$ local internal disparity variance

$\sigma_{\mathrm{Glob}}^{2}$ global internal disparity variance

$\sigma_{\mathrm{Int}}^{2}$ internal disparity variance

$\sigma_{\mathrm{Ext}}^{2}$ external disparity variance

$N\left( \sigma_{\mathrm{Loc}}^{2} \right)$ local internal disparity noise

$N\left( \sigma_{\mathrm{Glob}}^{2} \right)$ global internal disparity noise

$N\left( \sigma_{\mathrm{Int}}^{2} \right)$ internal disparity noise

$N\left( \sigma_{\mathrm{Ext}}^{2} \right)$ external disparity noise

$R_{+}$ response to uncrossed absolute disparity

$R_{-}$ response to crossed absolute disparity

$D_{\min}^{\mathrm{Abs}}$ absolute minimum disparity threshold

$D_{\min}^{\mathrm{Rel}}$ relative minimum disparity threshold

$d_{\pm}$ relative disparity between the top (uncrossed) and bottom (crossed) halves

$d_{\mp}$ relative disparity between the top (crossed) and bottom (uncrossed) halves

$R_{\pm}$ response to relative disparity $d_{\pm}$

$R_{\mp}$ response to relative disparity $d_{\mp}$

$R_{N}$ response to the interval only with noise

$I_{jL}$ luminance profile of jth Gabor patch presented in the left eye

$I_{jR}$ luminance profile of jth Gabor patch presented in the right eye

*d* stimulus disparity

ω spatial frequency of a Gabor patch

σ standard deviation of a Gabor patch

$m_{L}$ contrast of Gabor patches presented in the left eye

$m_{R}$ contrast of Gabor patches presented in the right eye

*x*, *y* two-dimensional index variables of the local positions of retinal inputs

*x_j_*, *y_j_* two-dimensional index of jth paired Gabor patches

$\theta_{j}$ phase of jth paired Gabor patches

$n_{jx}$ horizontal disparity random fluctuation of jth paired Gabor patches

$n_{jy}$ vertical disparity random fluctuation of jth paired Gabor patches

K the number of model parameters

$\nu$ the number of degrees of freedom

AIC Akaike Information Criterion

AICc Akaike Information Criterion with a correction for finite sample sizes

Aw Akaike weight

$\chi^{2}$ chi square, the residual sum of square in the least squares fitting

$\chi^{2}/\nu$ reduced chi square

1 Burnham, K. P. & Anderson, D. *Model selection and multi-model inference: a practical information-theoretic approach (2nd ed.)*. (Springer-Verlag, 2002).
